# Complete response for advanced liver cancer during sorafenib therapy: Case Report

**DOI:** 10.1186/1471-230X-11-4

**Published:** 2011-01-17

**Authors:** Rodolfo Sacco, Irene Bargellini, Giannelli Gianluigi, Marco Bertini, Elena Bozzi, Emanuele Altomare, Valentina Battaglia, Antonio Romano, Michele Bertoni, Alfonso Capria, Giampaolo Bresci, Carlo Bartolozzi

**Affiliations:** 1 Gastroenterology Department, Pisa University Hospital, Via Paradisa 2, 56124 Pisa, Italy; 2 Department of Diagnostic and Interventional Radiology, Pisa University Hospital, Via Paradisa 2, 56124 Pisa, Italy; 3 Institute of Internal Medicine, University of Foggia, Viale Pinto, 71100 Foggia, Italy; 4 Department of Internal Medicine, Immunology and Infectious Diseases, Section of Internal Medicine, University of Bari, 70124 Bari, Italy

## Abstract

**Background:**

Hepatocellular carcinoma (HCC) is the fifth most common neoplasia in the world. In the past, treatment of advanced HCC with conventional antineoplastic drugs did not result in satisfactory outcomes: recently, in this patient population the oral multikinase inhibitor sorafenib has been able to induce a statistically significant improvement of overall survival. Similarly to other anti-angiogenic drugs employed in other tumour types, also sorafenib seldom induces the dimensional tumour shrinking usually observed with conventional cytotoxic drugs: data gathered from studies carried out with sorafenib and other competitors under development do not report any complete response in HCV-induced HCC.

**Case presentation:**

An 84-year old man with a long-lasting history of chronic HCV hepatitis was referred to our Institution for an ultrasonography investigation of a focal hepatic lesion. To better characterize the liver disease and clearly define the diagnosis of the focal hepatic lesion, the patient was hospitalized in our department. Laboratory and instrumental investigations confirmed the clinical picture of HCV-related liver cirrhosis and identified a hepatic lesion of about 6 cm featuring infiltrating HCC with thrombosis of the portal trunk. Due to the advanced stage of the disease, therapy with sorafenib 400 mg bid was started. Right from one month after the treatment was started, a reduction of alpha-fetoprotein level was observed which, by the third month, turned down within the normal limits. In addition the CT scan showed 50% reduction of the neoplastic lesion along with canalization of the portal trunk. At the sixth month the normalization of the alpha-fetoprotein level at the lower limit of normality was confirmed and the MRI showed complete disappearance of the neoplasia. In addition a reduction of a metallo-proteinase serum level was obserdved. At the twelfth month a further MRI confirmed complete response had been maintained. At present the patient is in a follow-up program to evaluate the duration of the complete response.

**Conclusions:**

This case is worth mentioning since, to the best of our knowledge, it represents the first evidence of complete response to sorafenib in an elderly patient with advanced HCV-related HCC.

## Background

Hepatocellular carcinoma (HCC) is the fifth most common neoplasia worldwide with an incidence of about 620,000 new cases per year, and represents the third most common cancer-related cause of death. Even though it is particularly widespread in Asia and Africa, an increase of incidence has occurred in the last years also in the Western countries[1]. Five-year survival from diagnosis, strictly related to the stage of disease, has been estimated between 48% and 75%[2-4]. In the past, the treatment of advanced HCC with conventional antineoplastic drugs did not result in satisfactory outcomes[5]. In the recent years, however, advances achieved in the knowledge of molecular mechanisms underlying the growth of HCC led to the development of new entities useful for the treatment of hyper-vascularized tumours such as HCC. In this scenario, the oral multikinase inhibitor sorafenib is the only drug which is in two phase III pivotal, placebo-controlled clinical trials undertaken in patients with advanced HCC in the Western countries and in the Asia-Pacific area has been able to induce a statistically significant improvement of overall survival [6-8]. Similarly to other anti-angiogenic drugs, also sorafenib seldom induces the dimensional tumour shrinking usually observed in tumours responding to conventional cytotoxic drugs: data gathered from studies carried out with sorafenib and other competitors under development do not report any complete response in Hepatitis C Virus (HCV)-induced HCC.

## Case Presentation

In November 2009, an 84-year old patient was referred to our unit to ultrasonographically characterize a focal liver lesion of about 6 cm with thrombosis of the left portal trunk (PVT). The patient was in good shape (ECOG PS 0) with a history of chronic HCV-related hepatopathy clinically-functionally compensated. The aetiological frame carried out in our Unit confirmed the diagnosis of chronic hepatopathy evolving towards cirrhosis due to chronic HCV infection. Hepatic synthesis was well retained without clinical signs of liver impairment (CHILD A5). The following parameters turned out altered: AFP level 353 ng/ml (nv <7); CA 19-9 45.8 U/ml (nv <39); AST 99 (nv <40 U/L), ALT 142 (nv <41 U/l); platelet count 126/L (nv 150-400). Kidney function was within the normal limits.

**Figure 1:**
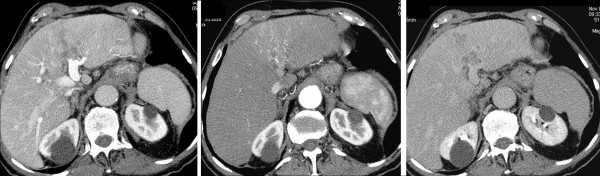
**Baseline CT examination: 1a, arterious phase; 1b, portal phase; 1c, late phase**. Between III and IV hepatic segment is possible to observe the presence of a solid lesion with poorly defined margins and infiltrative nature; the lesion is hyperdense in arterial phase and hypodense in the portal phase and late, and it invades the left main portal vein which is thrombosed.

Contrast ultrasonography substantiated the evidence elsewhere detected. Multidetector abdomen CT scan (Light Speed Plus, GE Medical Systems, Milwaukee, USA) carried out using three-phase system following administration of iodine contrast medium (Iomeron 400, Bracco, Milan, Italy) confirmed the presence of a lesion of about 6 cm located between the hepatic segments IV and III characterized by indistinct margins and infiltrating, with wash-in in arterial phase and wash-out in late phase, typical of HCC. The lesion spread from the liver dome and from the margin of the Glisson capsule to the anterior area of the left portal trunk and its branch to segment III which were clotted. Splenomegaly (16 cm DL) and gallbladder microlithiasis were also pointed out (Figure 1). No signs of ascitis were observed. Chest CT did not detect any metastatic lesion.

Upper digestive tract endoscopy did not show signs of esophageal varix. The cardiac function evaluation (ECG, echocardiography and clinical) excluded any symptom of heart failure.

On the basis of this picture, a diagnosis of advanced HCC was made (BCLC C).

Being impossible to utilize loco-regional therapy, a treatment with sorafenib tablets with a dose of 400 mg bid was started.

The outcome of the therapy can be summarized as follows:

*1st assessment (after one month) *- decrease of AFP level to 113 ng/ml and conversion of CA-19-9 to normal values (34 U/ml).

**Figure 2:**
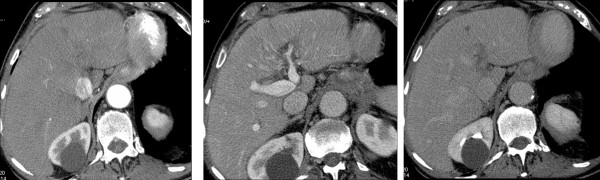
**three-months follow-up with CT: 2a, arterious phase; 2b, portal phase; 2c, late phase**. The three months CT scan document an apparent reduction in the size of lesion, which is associated with partial recanalization of the left portal branch.

*2nd assessment (after three months) *- further decrease of AFP value to 6.6 ng/ml. The three-phase multidetector CT (Light Speed Plus) with contrast medium highlighted a marked dimensional reduction of infiltrating focality at the segment III-IV (from 6 cm to 3 cm) (Figure 2). Residual disease was predominantly localized at the segment IV of the liver in the upper site area of the left portal trunk which, in any case, was re-canalized as compared to the previous assessment. Also the portal trunk of the (delete) segment III appeared re-canalized. No evidence of new focalities and continuous absence of ascites have been pointed out.

**Figure 3:**
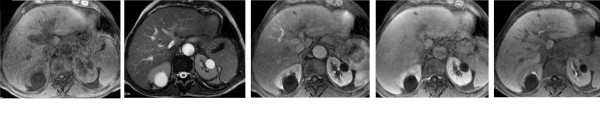
**Six months follow up with MRI before and after injection of Primovist: 3a, T1-weighted; 3b, T2-weighted; 3c, arterial phase; 3d, portal venous phase; e, hepatobiliary phase**. At six months of treatment, the liver injury is no longer recognizable at MRI examination and the left portal branch is fully recanalized, as with almost complete response to treatment.

*3rd assessment (after six months) *- After six months of treatment with sorafenib the AFP value accounted for 2.3 ng/ml. The MRI (Signa HDx; GE Healthcare, Milwaukee, WI) with liver-specific contrast medium (Primovist, Bayer Schering) (figure 3) did not identify at all the lesion at segment III and IV. The portal trunk was pervious and no other hypervascular lesions in the liver parenchyma were seen. The parameters of liver function were unchanged. In agreement with RECIST evaluation criteria, the disappearance of all the target lesions led to classify a complete response.

**Figure 4:**
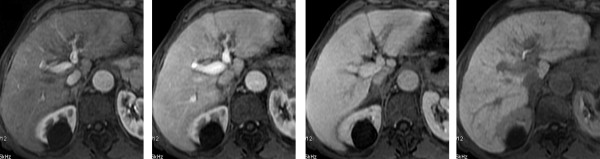
**Twelve-months follow up with MRI before and after after injection of Primovist: 5a, T1-weighted; 5b, T2-weighted**:On baseline T1w.i and T2w.i no sign of changes of signal intensity within the liver parenchyma is appreciable, not even at the level of the previous lesion of segment III.

**Figure 5:**
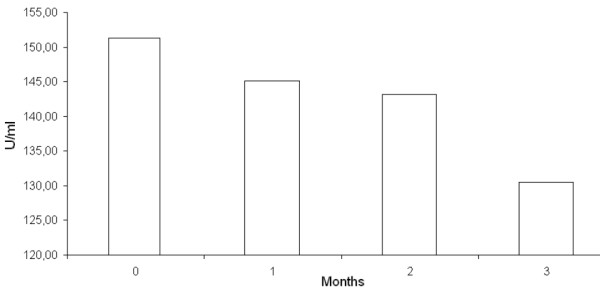
**Twelve-months follow up with MRI before and after after injection of Primovist: 6a, arterial phase; 6b portal venous phase; 6c portal venous phase delayed; 6d hepatobiliary phase**. On post contrastographic dynamic study, there is no focal enhancement on arterial phase 6a, nor signs of portal branches invasion on portal venous phase 6b, nor areas of hypointensity on delayed phase 6c. On hepatobiliary phase 6d, homogeneous enhancement of the previous neoplastic parenchyma of segment III is now appreciable.

*4rd assessment (after twelve months) *- By the twelfth month the patient performed a new MRI with liver-specific contrast medium (Primovist, Bayer Schering) (figure 4 and 5). This confirms CR had been maintained. Treatment with sorafenib continues and a follow-up program to evaluate the duration of complete response is in progress.

The safety aspect of the treatment with sorafenib was acceptable and manageable: the only adverse events observed consisted of cutaneous rash requiring treatment with urea cream, and mild-moderate asthenia not negatively interfering with patient's daily activities.

**Figure 6:**
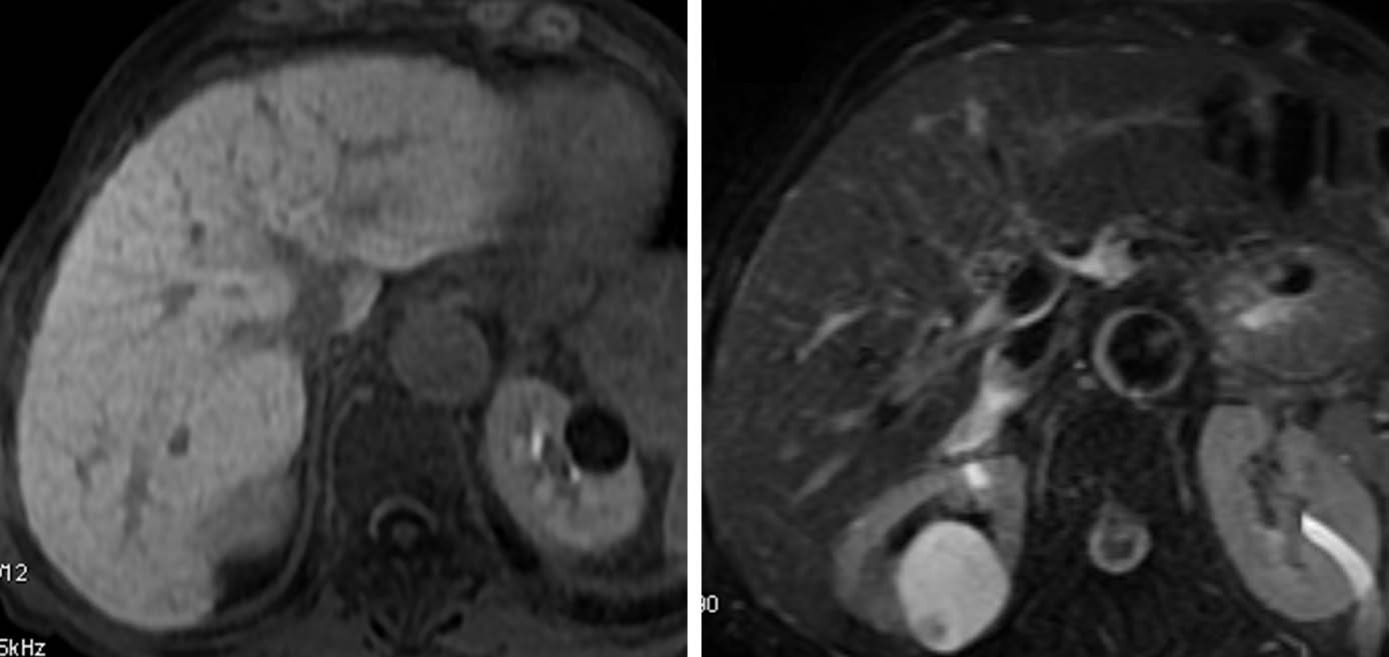
**Analysis of metallo-proteinase 9 serum level**: A clear reduction of marker serum level is observed after three months of treatment

Lastly the patient has been addressed to a program aiming at correlating the serum levels of metallo-proteinase 9 with the disease course. Analysis available on the third month, showed a marked reduction of metallo-proteinase 9 level during the treatment with sorafenib. (Figure 6).

## Conclusions

The present case deals with a rapid and complete response, extensively supported by imaging and by the evaluation of tumour markers, likely occurred in a patient with advanced HCC (BCLC stage C) treated with sorafenib.

The efficacy of sorafenib in this tumour has already been demonstrated in two pivotal multicentre phase III placebo-controlled clinical trials. The first one (SHARP study), which accrued more than 600 patients with advanced HCC with cirrhosis (Child Pugh A) not previously treated with any systemic therapy, for the first time showed an advantage in terms of overall survival in patients with advanced HCC. Among patients treated with sorafenib, however, only seven (2%) were considered responders according to RECIST criteria and no complete response was recorded [6]. Similar considerations can be made as far as the second phase III clinical study carried out in the Asia Pacific Area is concerned [7].

To our knowledge, the above reported case represents the first example of complete response observed in an elderly patient with advanced HCC and concomitant HCV infection. So far, literature data reported a case of complete response to sorafenib in a patient with HCC due to hemochromatosis [9] and a reduction of a lung metastasis in a transplanted patient with HBV-related HCC [10]. Even though spontaneous regressions have been sometimes described in patients with HCC [11,12], the present case seems definitely unusual since it occurred in an elderly patient with infiltrating disease and portal thrombosis which, as it is well-known, represent the main negative prognostic factors for HCC. In our experience, the 84-year old patient showed a good tolerability to treatment and the incidence and severity of adverse event did not differ from data reported in the pivotal studies carried out in HCC. Additional information supporting the use of sorafenib in elderly patients can be found in studies evaluating sorafenib in renal carcinoma[13,14] which indicate that in these patients a benefit can be achieved as in the younger ones provided that a careful monitoring of adverse event is done[15]

Another interesting point is the re-canalization of the portal vein. Portal thrombosis is a serious complication due to cirrhosis whose frequency increases in patients with HCC. This event, considered the main negative prognostic factor in patients with HCC, represents the final outcome ensuing from the direct invasion of the portal vein and the production by neoplastic cells of pro-coagulative cytokines [16]. In our case, a significant reduction of the infiltrating disease leading to re-canalization of the portal vein has been observed. HCC is a hypervascular tumor. Literature data reports that the loss of vascularity indicates tumor necrosis: reduction or loss of vascularity after therapy might be an important measure of patient response to the treatment [17].. It has been hypothesized that sorafenib could exert a dual activity: the first one directly on neoplastic cells and the second one trough a modulation of pro-thrombotic cytokines [16].

In fact one report has suggested that Vascular Endothelial Growth Factor (VEGF) may play an important role both in HCC angiogenesis, in portal thrombosis and its evolution [18].

Sorafenib could exert a beneficial effect on portal thrombosis through the inibition of VEGF pathway.

Since a relation between serum levels of metallo-proteinase 9 and pathogenetic mechanism underlying the HCC genesis has been recently hypotesized [19-22], this case has been inserted in a program aiming at verifying such relation.

Metalloproteinase-9 could be involved in neoangiogenesis processes and in tumour progression [20,23] and some in vitro studies seem to indicate that sorafenib could play a role in modulating the Metalloproteinase-9 expression through a down-regulation process [22]. The clear reduction of marker serum levels observed after three months of treatment seem to confirm the hypothesis that the reduction of these levels could be related to a lower angiogenesis, a lower invasiveness and a lower disease progression.

A limiting aspect of this case could lie in the lack of biopsy. According to the current guidelines, however, in presence of nodules larger than 2 cm showing a typical post-contrast pattern (wash-in in the arterial phase and wash-out in the late phase) biopsy is not strictly required for diagnosis of HCC. In our case, the diagnosis was based on the typical feature of a 6 cm lesion subsequently substantiated by the consistency of the two imaging findings [24,25]. Certainly a biopsy could have been helpful to get information on the molecular pattern of the disease and then on the possible interaction with sorafenib.

This surprising and unexpected outcome raises some questions and could be a matter of debate. For instance, it could be assumed that in the tumour cells mutations or alterations of the genomic profile making them more sensitive to sorafenib could have occurred. If this hypothesis is trustworthy, it represents a valid reason to boost new investigations aiming at studying in depth further genetic and molecular features to identify more selected, or even individual, patterns of response.

## Consent

Written informed consent was obtained from the patient for publication of this case report and any accompanying images. A copy of the written consent is available for review by the Editor-in-Chief of this journal.

## List of abbreviations

AFP: alpha-fetoprotein; ALT: alanine aminotransferase; AST: aspartate aminotransferase; CA-19-9: carbohydrate antigen 19-9; CT: computerazied tomography; HBV: hepatitis B virus; HCC: hepatocellular carcinoma; HCV: hepatitis C virus; MRI: magnetic resonance imaging; PTV: portal vein thrombosis; RECIST: response evaluation criteria in solid tumors; VEGF: Vascular Endothelial Growth Factor

## Competing interests

The authors declare that they have no competing interests.

## Authors' contributions

RS took care of patient, collected and analyzed data, and wrote the manuscript; IB, EB, VB, performed and analyzed diagnostic imaging and contributed to discussion; GG evaluated plasmatic metallo-proteinase levels; MB, AR, MB, GB involved in follow-up of patient: EA, AC and CB coordinated the work group and contributed to discussion. The authors read and approved the final manuscript

## Author details

Rodolfo Sacco, Marco Bertini, Antonio Romano, Michele Bertoni, Alfonso Capria, Giampaolo Bresci: Gastroenterology Department, Pisa University Hospital, Via Paradisa 2, 56124 Pisa, Italy.

Irene Bargellini, Elena Bozzi, Valentina Battaglia, Carlo Bartolozzi: Department of Diagnostic and Interventional Radiology, Pisa University Hospital, Via Paradisa 2, 56124 Pisa, Italy

Rodolfo Sacco, Emanuele Altomare: Institute of Internal Medicine, University of Foggia, Viale Pinto, 71100 Foggia, Italy.

Giannelli Gianluigi: Department of Internal Medicine, Immunology and Infectious Diseases, Section of Internal Medicine, University of Bari, 70124 Bari, Italy.

## Pre-publication history

The pre-publication history for this paper can be accessed here:

http://www.biomedcentral.com/1471-230X/11/4/prepub
